# Data on CaO and eggshell catalysts used for biodiesel production

**DOI:** 10.1016/j.dib.2018.06.028

**Published:** 2018-06-21

**Authors:** Ayoola A. Ayodeji, Ojewumi E. Modupe, Babalola Rasheed, James M. Ayodele

**Affiliations:** a Chemical Engineering Department, Covenant University, Ota, Nigeria; b Chemical/Petrochemical Engineering Department, Akwa Ibom State University, Nigeria

**Keywords:** Biodiesel, Calcined eggshell, Catalyst

## Abstract

This research investigated the production of biodiesel from soybean oil (transesterification process) using pure calcium oxide and calcium oxide obtained from eggshell as heterogeneous catalysts. Uncalcined eggshell and calcined eggshell catalysts produced were analysed using XRF and XRD spectrometers. The processing parameters considered during the transesterification of the soybean were methanol/oil mole ratio, catalyst concentration and reaction time and their effects on biodiesel yield were evaluated. Reaction temperature of 60 °C and stirring rate of 450 rpm (revolution per minute) were kept constant. As a result of calcination, XRF analysis revealed an increase in CaO percentage composition of eggshell catalyst from 96% to 97%. Also, the biodiesel yields obtained revealed similar performance patterns for both the calcined eggshell catalyst and the pure CaO catalyst.

**Specification Table**

**Table:** 

Subject area	*Materials Science Engineering*
More specific subject area	*Renewable Energy*
Type of data	*Table, image*
How data was acquired	The physico-chemical characteristics (chemical compositions) of the uncalcined eggshell, calcined eggshell and conventional CaO catalysts were determined using XRF and XRD spectroscopy principles. With the aid of Minitab software, data on the yields of biodiesel produced was generated from the transesterification of soybean oil, using the conventional CaO and calcined eggshell catalysts. Data on biodiesel properties was obtained from the various simple laboratory tests carried out.
Data format	Raw, Analyzed
Experimental factors	Processing parameters considered in the transesterification of soybean oil were methanol/oil mole ratio, catalyst concentration and reaction time.
Experimental features	XRD and XRF analyses that were carried out involved investigating the elemental composition of the conventional CaO, uncalcined and calcined eggshell catalysts. During the transesterification process of soybean oil, methanol/oil mole ratio of 8–14, catalyst concentration of 3–5 wt/wt% and reaction time of 1–3 h were considered. Reaction temperature of 60 °C and a stirring rate of 450 rpm were kept constant.
Data source location	Chemical Engineering Department, Covenant University, Ota, Nigeria and Metallurgical and Chemical Engineering Department, Amadu Bello University, Zaria, Nigeria.
Data accessibility	Data are available within this article

**Value of the data**

• The data on biodiesel production can be modelled to establish correlation between the operating parameters and the yields of biodiesel.
• Egg shell data obtained shows that the calcined eggshell resulted in an increase in CaO content.
• The data obtained from the transesterification of soybean oil can be interpolated to determine optimal conditions for the production of biodiesel.
• The data reveals that calcined eggshell catalyst is a potential source of CaO heterogeneous catalyst that can be used in the place of the conventional CaO catalyst during oil transesterification. A positive step in overcoming environmental pollution associated with the wrong disposal of waste egg shells.

## 1 Data

**Fig. 1:**
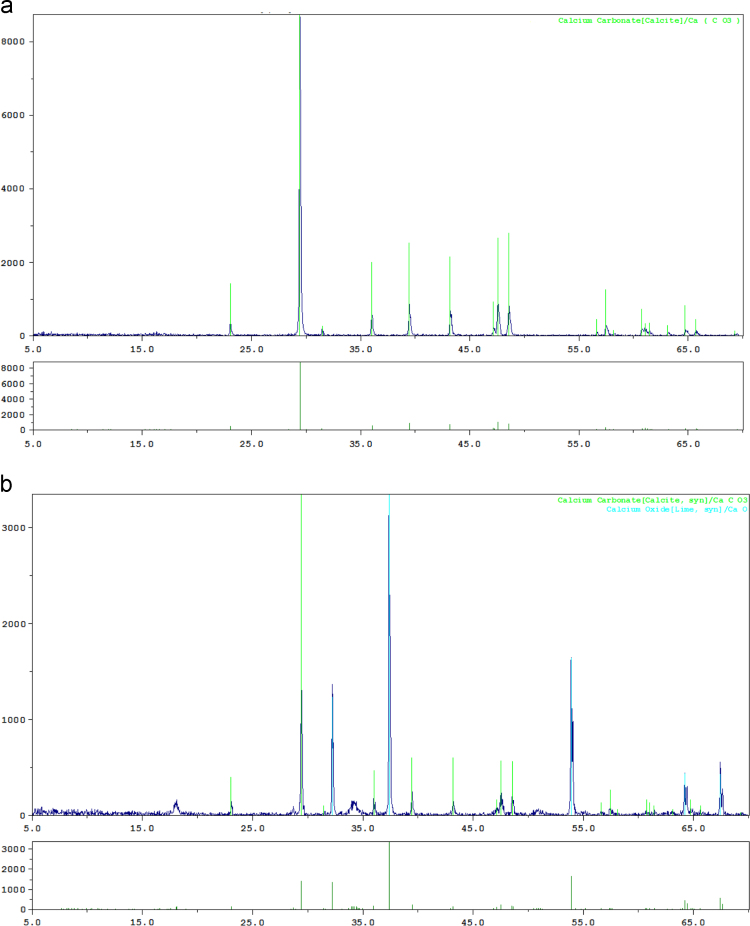
XRD spectrometry of: (a) uncalcined eggshell, (b) calcined eggshell catalyst.

**Fig. 2:**
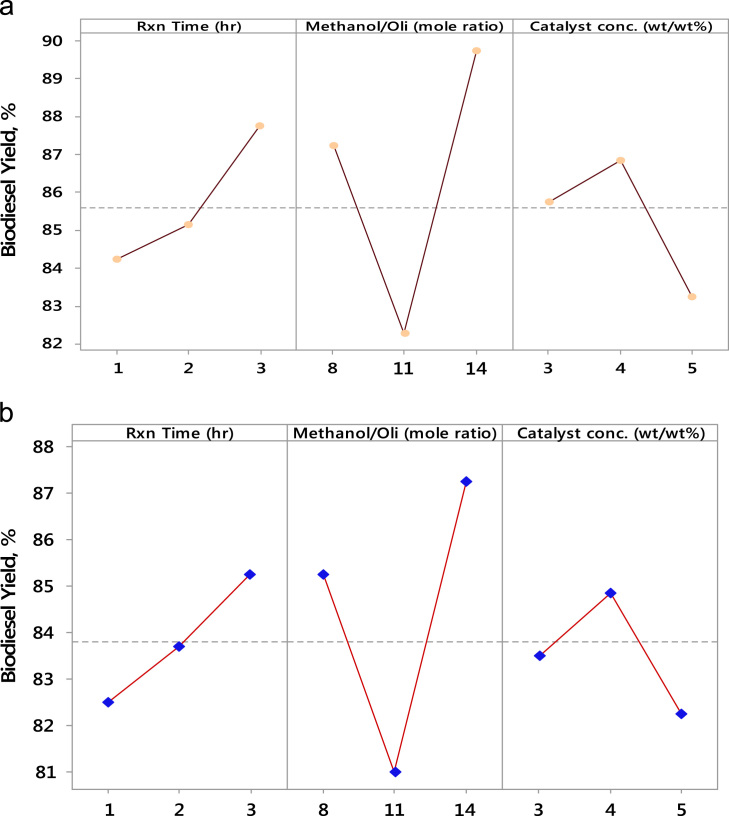
Main effects of the process variables (a) CaO catalyst and (b) eggshell catalyst.

**Fig. 3:**
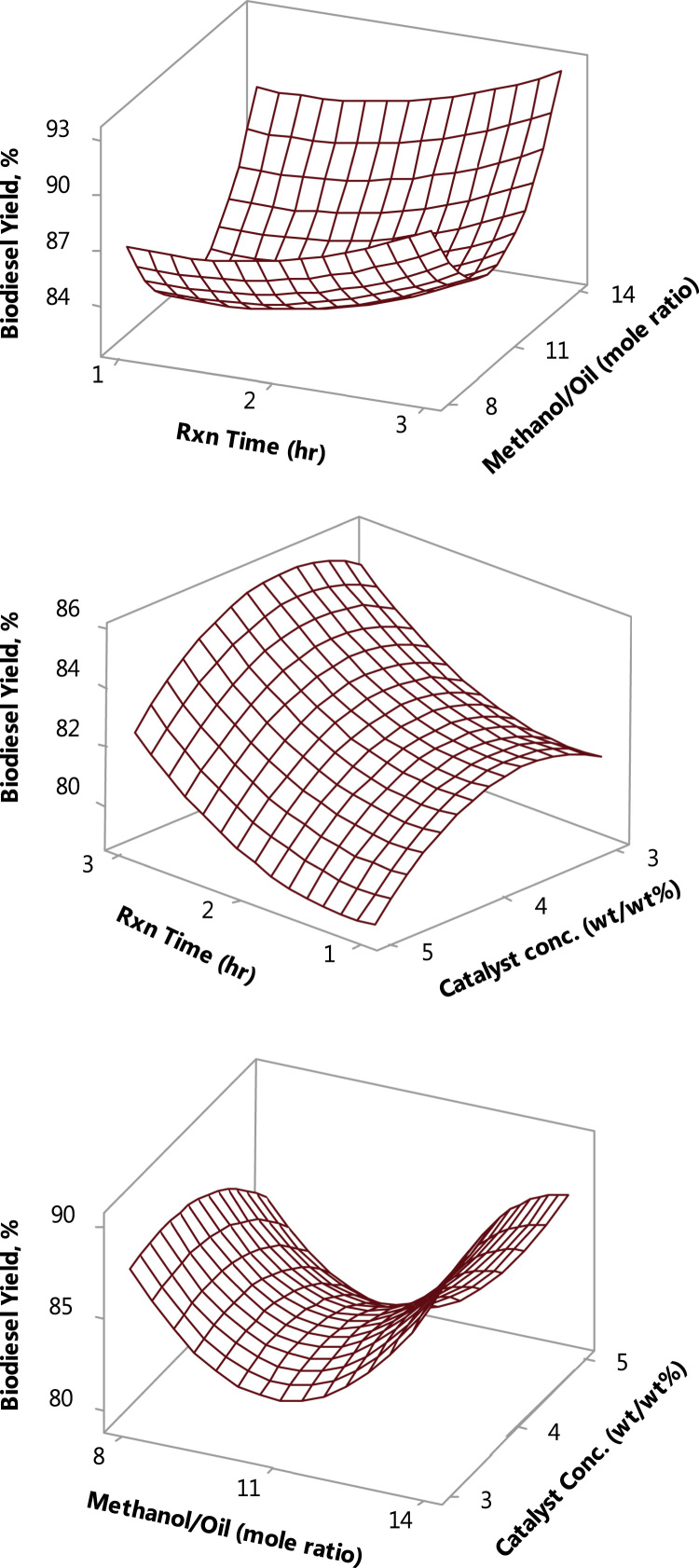
Interactive effects of the process variables, using conventional CaO catalyst.

**Fig. 4:**
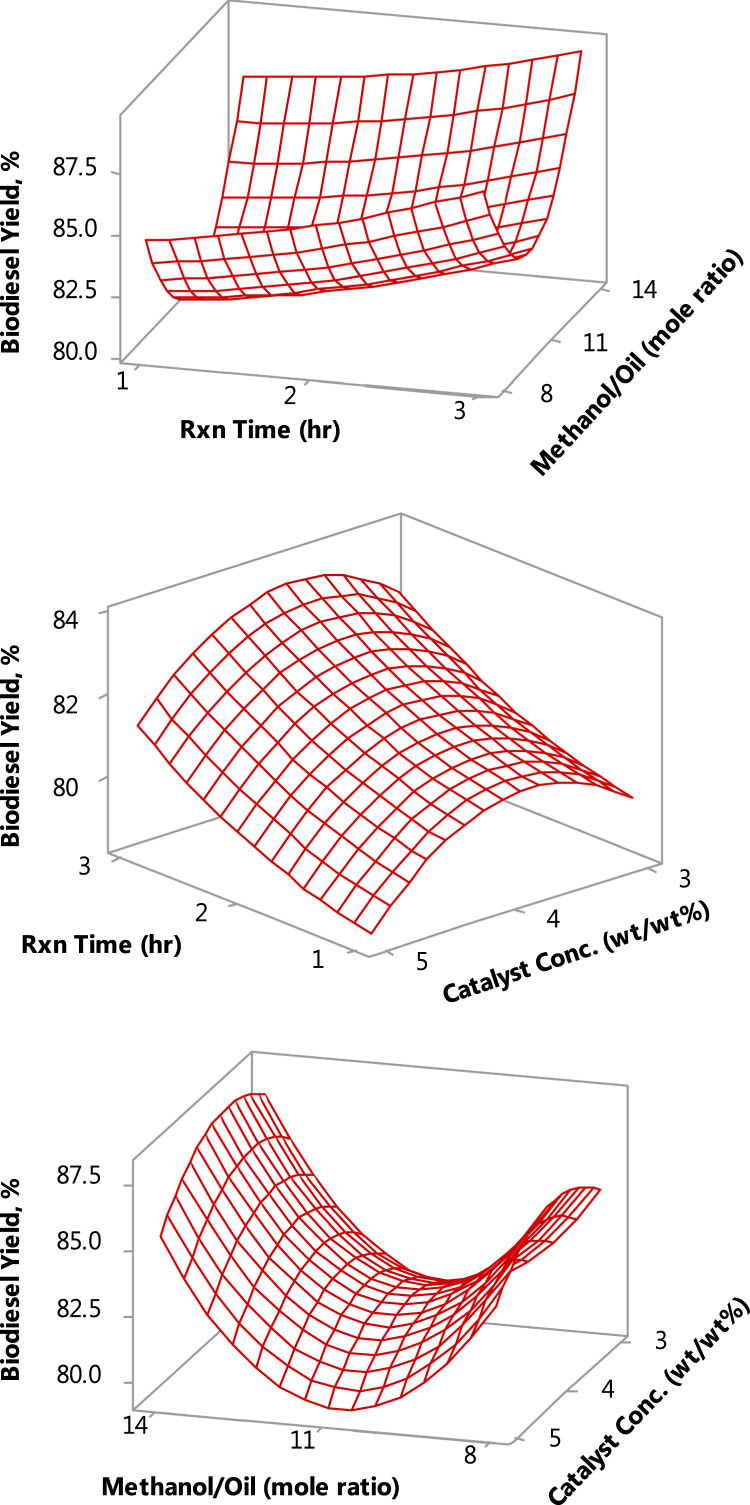
Interactive effects of the process variables, using eggshell catalyst.

XRF analysis of CaO, uncalcined eggshell catalyst and calcined eggshell catalyst were presented in Table 1. XRD analysis of uncalcined eggshell (mostly CaCO_3_) and calcined eggshell catalyst (mostly CaO) were presented in Fig. 1. Table 2 reveals the biodiesel yield data obtained from the transesterification process, using the conventional CaO and calcined eggshell catalysts separately. The main effects of the process variables during transesterification are shown in Fig. 2. The interactive effects of the process variables during transesterification, using the conventional CaO catalyst are shown in Fig. 3, while the interactive effects of the process variables during transesterification, using eggshell catalyst are shown in Fig. 4. Table 3 reveals the properties of the biodiesel produced.

**Table 1:** XRF spectrometry of the conventional CaO, Uncalcined and calcined eggshell catalysts.

**Compound**	**Chemical Composition of the Catalyst (wt%)**		
	Conventional	Uncalcined	Calcined
	CaO	Eggshells	Eggshells
MgO	–	0.531	0.544
Al_2_O_3_	–	0.353	0.255
SiO_2_	–	1.504	1.266
P_2_O_5_	–	0.283	0.316
SO_3_	–	0.559	0.123
K_2_O	–	0.118	0.103
CaO	99.230	96.131	97.080
Mn_2_O_3_	–	0.003	0.000
Fe_2_O_3_	0.110	0.017	0.013
SrO	0.120	0.305	0.263
ZrO_2_	0.010	–	–
BaO	0.240	–	–
La_2_O_3_	0.130	–	–
CeO_2_	0.040	–	–
Pr_2_O_3_	0.040	–	–
Yb_2_O_3_	0.050	–	–

**Table 2:** Experimental design and biodiesel yields obtained from the transesterification process.

Rxn time	Methanol/Oil	Catalyst conc.	% Biodiesel yield	% Biodiesel yield
(h)	(mole ratio)	(wt/wt%)	(using CaO catalyst)	(using eggshell catalyst)
2	8	3	88	85
3	14	4	94	91
1	8	4	86	83
1	11	3	82	81
2	11	4	83	82
2	8	5	84	84
2	14	5	87	85
3	11	5	82	80
3	8	4	91	89
2	11	4	82	81
1	14	4	89	86
1	11	5	80	80
2	14	3	89	87
2	11	4	83	82
3	11	3	84	81

**Table 3:** Properties of soybean biodiesel produced.

Sample	Density @ 25 °C (g/cm^3^)	Pour Point (°C)	Flash point (°C)	Viscosity @ 40 °C (mm^2^/s)	Water content (%)	Acid value (mg KOH/g)
Soybean biodiesel	0.8900	−2	148	4.32	0.005	0.040

## 2 Experimental design, materials and methods

Response surface experimental design (Box-Behnken method, Minitab 17 software) was employed to investigate the effects of variation of methanol-oil mole ratio, catalyst concentration and reaction time on biodiesel yield, using a similar approach published in Ref. [1]. Materials used include methanol (99% purity, Qualikems, India), CaO (99.2%, Romil Ltd UK), waste eggshell and soybean oil. Equipment used include XRD and XRF spectrometers.

Waste eggshells were first carefully washed in clean water to remove sand, tissue, and other impurity present. And then dried in oven (110 °C for 40 min). The thin lining in the inner part of the shells were then carefully removed. The eggshells were then crushed (using electrical crushing machine) and grinded into fine particles. Fine particle size of 80 µm was obtained through sieve analysis. The powder obtained was calcined at temperature of 850 °C in an electric furnace for 4 h to ensure complete transformation of eggshell rich in CaCO_3_ to catalyst rich in CaO [2], [3], [4].

Determination of the elemental composition of uncalcined and calcined eggshell catalysts involved the use of XFD and XRF analysis. Phillips 1404 XRF Wavelength Disperse Spectrometer coupled with X-ray tube and a Rh anode (X-rays source) having HVPS 60 kV, 7.0 mA, a LN_2_ cooled Si(Li) detector with a resolution of 131 eV at, Mn Kα (5.9 keV) X-ray and a 6-sample turret (that permits the mounting and analyzing of 6 samples at a time) was used. XRF spectrometer operation was based on the emission of the excited elemental components of the given sample through the bombarding of the sample with high energy X-rays [1].

For the uncalcined eggshell, main peak was observed at 2*θ*=29.0°, other peaks were noticed at 2*θ*=36.0°, 39.0°, 44.0°, 47.0° and 48.0° (Fig. 1a). These peak values were the characteristics of CaCO_3_. And the peak for the calcined eggshell catalyst were measured at 2*θ*=29.0°, 32.5°, 47.5° and 54.0°, the main peak values characteristics of calcium oxide (Fig. 1b).

Soybean biodiesel production involved transesterification process as reported in Ref. [5]. Comparatively, the result of biodiesel yield revealed similar behavioral pattern between the conventional CaO and calcined eggshell catalysts. Properties of the soybean biodiesel produced were established, using simple laboratory equipment.
