# Supplementary material for: Data on CaO and eggshell catalysts used for biodiesel production
Source: Data Brief. 2018 Jun 21;19:1466–73. doi: 10.1016/j.dib.2018.06.028 (PMC6141156; doi:10.1016/j.dib.2018.06.028)
Supplement: Supplementary file 1 — Supplementary material [file mmc1.docx]

Chemical Engineering Department

Covenant University,

Nigeria

April 11, 2018

Dear Editor,

Data In Brief

**COVER LETTER**

The research article titled “**Data on CaO and Eggshell Catalysts Used for Biodiesel Production ”** is being submitted for publication in *Data In Brief*.

On behalf of all the authors, I declare that the manuscript (authored by Ayoola A. Ayodeji, Ojewumi E. Modupe, Babalola Rasheed, James M. Ayodele) has not been published in another publication and it is not being submitted simultaneously to another journal.

Forwarded for submission are the manuscript and conflict of interest form.

Thank you

Yours faithfully,

**Ayoola A. Ayodeji**

***Corresponding Author***
